# Toxin exposure and HLA alleles determine serum antibody binding to toxic shock syndrome toxin 1 (TSST-1) of *Staphylococcus aureus*

**DOI:** 10.3389/fimmu.2023.1229562

**Published:** 2023-09-04

**Authors:** Stefan Weiss, Silva Holtfreter, Tanja C. Meyer, Frieder Schmiedeke, Clemens Cammann, Marcus Dörr, Stephan B. Felix, Hans J. Grabe, Georg Homuth, Christian Kohler, Cedric Mahncke, Stephan Michalik, Matthias Nauck, Nele Friedrich, Stefanie Samietz, Henry Völzke, Uwe Völker, Barbara M. Bröker

**Affiliations:** ^1^ Interfaculty Institute for Genetics and Functional Genomics, University Medicine Greifswald, Greifswald, Germany; ^2^ German Centre for Cardiovascular Research (DZHK), Partner Site Greifswald, Greifswald, Germany; ^3^ Institute of Immunology, University Medicine Greifswald, Greifswald, Germany; ^4^ Friedrich Loeffler Institute of Medical Microbiology, University Medicine Greifswald, Greifswald, Germany; ^5^ Department of Internal Medicine B, University Medicine Greifswald, Greifswald, Germany; ^6^ Department of Psychatry and Psychotherapy, University Medicine Greifswald, Greifswald, Germany; ^7^ Institute of Clinical Chemistry and Laboratory Medicine, University Medicine Greifswald, Greifswald, Germany; ^8^ Department of Prosthetic Dentistry, Gerodontology and Biomaterials, University Medicine Greifswald, Greifswald, Germany; ^9^ Institute for Community Medicine, University Medicine Greifswald, Greifswald, Germany

**Keywords:** *Staphylococcus aureus*, toxic shock syndrome, TSST-1, superantigen, GWA, HLA, MHC, antibody

## Abstract

Life-threatening toxic shock syndrome is often caused by the superantigen toxic shock syndrome toxin-1 (TSST-1) produced by *Staphylococcus aureus*. A well-known risk factor is the lack of neutralizing antibodies. To identify determinants of the anti-TSST-1 antibody response, we examined 976 participants of the German population-based epidemiological Study of Health in Pomerania (SHIP-TREND-0). We measured anti-TSST-1 antibody levels, analyzed the colonization with TSST-1-encoding *S. aureus* strains, and performed a genome-wide association analysis of genetic risk factors. TSST-1-specific serum IgG levels varied over a range of 4.2 logs and were elevated by a factor of 12.3 upon nasal colonization with TSST-1-encoding *S. aureus*. Moreover, the anti-TSST-1 antibody levels were strongly associated with HLA class II gene loci. HLA-DRB1*03:01 and HLA-DQB1*02:01 were positively, and HLA-DRB1*01:01 as well as HLA-DQB1*05:01 negatively associated with the anti-TSST-1 antibody levels. Thus, both toxin exposure and HLA alleles affect the human antibody response to TSST-1.

## 1 Introduction

Toxic shock syndrome (TSS) is an acute live-threatening toxin-mediated disease caused by the pathobiont *Staphylococcus (S.) aureus.* It is characterized by fever, scarlet-like rash, multi-organ dysfunction, hypotension, and shock (1, 2). In 1980, the use of highly absorbent tampons in the United States caused an outbreak of menstrual TSS (mTSS) in young women (3). By eliminating highly absorbent materials and providing education on proper usage, the incidence of mTSS dropped from approximately 13.7 in 1980 to 1 per 100,000 menstruating women in 1986 (4, 5). Non-menstrual TSS (nmTSS) is caused by *S. aureus* infections at other foci and is reported from both males and females. TSS is strongly linked to toxic shock syndrome toxin-1 (TSST-1)-producing *S. aureus* strains, and a lack of anti-TSST-1 antibodies is a well-known risk factor for TSS (4, 6). However, determinants of low antibody titers against TSST-1 are still largely unknown.

The staphylococcal superantigen (SAg) TSST-1 is the major cause of TSS. The TSST-1-encoding gene *tst* is detected in 89-100% of mTSS isolates as well as in approximately 50% of nmTSS isolates (4, 7, 8). TSST-1, but not staphylococcal enterotoxins, causes TSS after vaginal application in rabbits (9). Recent data point to a direct activation of vaginal epithelial cells via CD40, which leads to chemokine release and subsequent recruitment of immune cells (10). SAg genes including *tst* are encoded by mobile genetic elements, rendering the SAg gene profile of *S. aureus* strains highly variable. The *tst* gene is located on staphylococcal pathogenicity islands (SaPI) and strongly linked to the wide-spread clonal complex (CC) 30 (1, 11, 12). Accordingly, CC30 isolates account for the majority of mTSS cases and were also frequently isolated from tampons recovered from a cohort of healthy women (13). nmTSS is frequently caused by other *S. aureus* lineages, which lack *tst* but harbor other SAg genes, including genes for the staphylococcal enterotoxins SEA, SEB, SEC and SED (1, 4).

SAgs are very potent T cell mitogens, inducing massive T cell proliferation and cytokine release. They circumvent conventional antigen recognition by cross-linking major histocompatibility complex class II (MHC II) molecules on antigen presenting cells (APCs) with T cell receptors (TCRs) on T cells (14). Each SAg binds to a certain subset of TCR Vβ elements, and therefore activate T cells irrespective of their antigen-specificity (15). TSST-1 activates TCR Vβ2-bearing T cells, which account for up to 10% of T cells in human peripheral blood, expanding them in fatal cases to up to 30-70% (16). Superantigenic activation of T cells and APCs (e.g. macrophages) causes strong activation of NFκB and massive cytokine release; predominantly interleukin (IL)-2, tumor necrosis factor (TNF-)α and interferon (IFN-)γ by T cells as well as TNFα, IL-1β and IL-6 by monocytes (2, 14). This causes fever, and generalized capillary leakage and hypotension, ultimately resulting in shock and organ failure.

SAgs are highly immunogenic and antibodies against TSST-1 as well as the staphylococcal enterotoxins are common in the general population (17–19). Seroconversion occurs in the course of *S. aureus* infection, e.g. sepsis, and endocarditis (20–22). The most frequent route of exposure to TSST-1, however, is nasal colonization. Around 20% of the population are persistently colonized with *S. aureus*, with the remainder likely being intermittently colonized (23). As CC30 ranks among the top 3 *S. aureus* lineages in population-based studies around the world, everyone likely gets exposed to the CC30-linked TSST-1 toxin during life (11, 24, 25). TSST-1 binding antibodies correlate with protection against TSS (6, 17, 18, 26). It was estimated that only 9-15% of young US American adults lack sufficient antibody titers against TSST-1 (i.e. have ELISA titers < 100) (27, 28). At the onset of mTSS, however, antibodies against TSST-1 were reported as lacking or low in up to 98% of the patients (29). Around half of these patients do not seroconvert over the course of disease (29). These numbers illustrate that a lack of anti-TSST-1 antibodies is a major risk factor for TSS.

In this study, we aimed to identify genetic determinants and other risk factors for low titers of anti-TSST-1 antibodies by studying a sample of 976 participants of the population-based Study of Health in Pomerania (SHIP-TREND-0). Here, we report that toxin exposure due to nasal colonization with *tst*-positive *S. aureus* strains as well as particular HLA alleles determine serum antibody levels against TSST-1.

## 2 Results

### 2.1 Recent exposure by nasal colonization enhances anti-TSST-1 antibody titers

**Figure 1:**
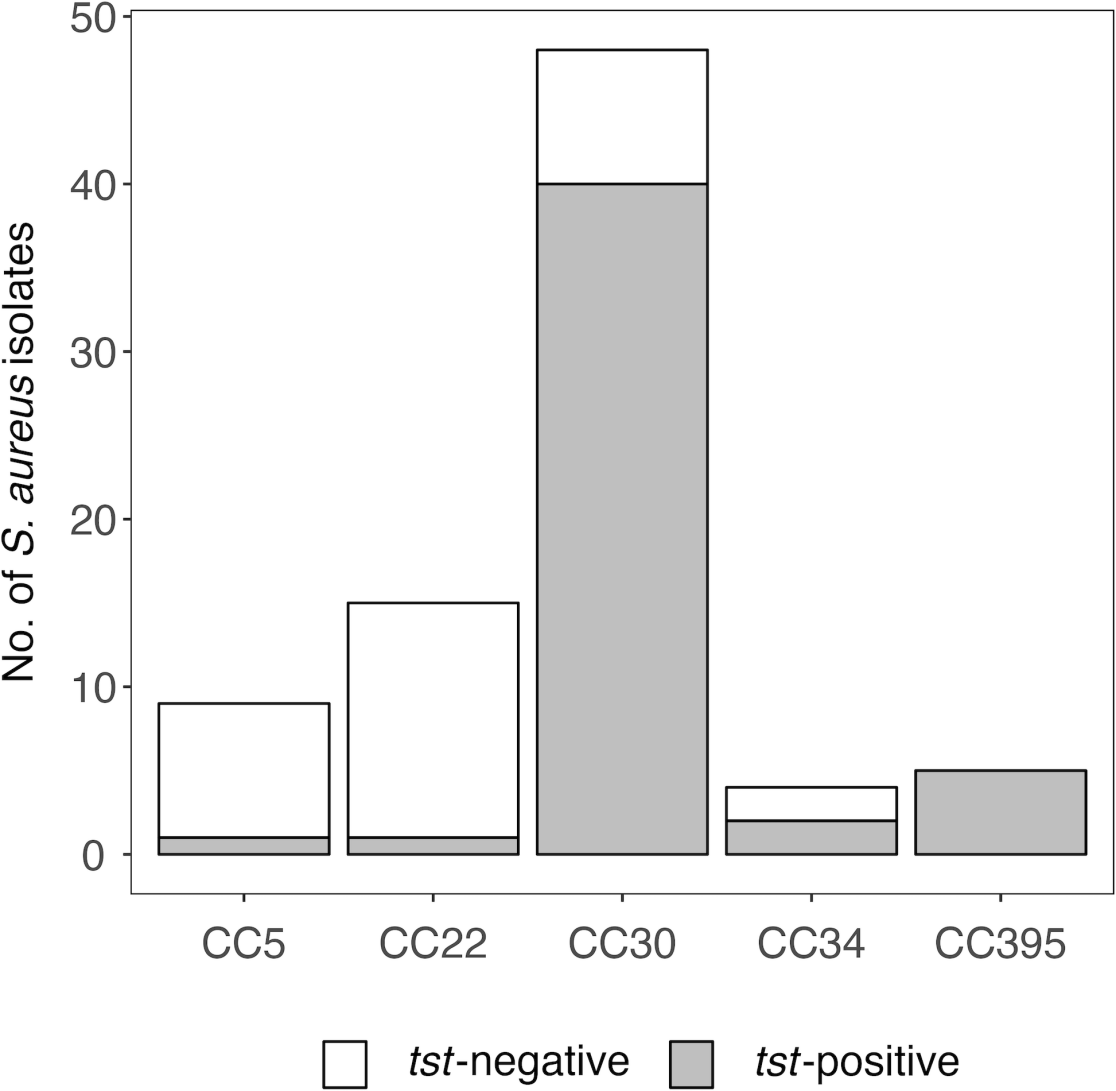
Prevalence of *tst* is linked to *S. aureus* clonal complexes. *S. aureus* nasal colonization was examined in the population-based study SHIP-TREND-0. *S. aureus* isolates were assigned to clonal complexes (CCs) based on *spa* typing (11). The gene *tst* was detected by multiplex PCR (11).

To identify determinants of the anti-TSST-1 antibody response, we examined a sub-sample of 976 participants in SHIP-TREND-0. For this study sample a complete data set was available, comprising the *S. aureus* colonization status, anti-TSST-1 antibody profiles, and genome-wide genotyping data for single-nucleotide variants (SNVs) (**Supplementary Table 1**) (11, 19, 30, 31). Of these individuals, 25.5% (249/976) were intranasally colonized with *S. aureus*. *Spa* typing and PCR data were available for 240 of these isolates. The lineage CC30 was most prevalent, accounting for 20.0% (48/240) of the *S. aureus* isolates. The SAg gene *tst* was detected in 20.4% (49/240) of the isolates. As previously reported (11), this SaPI-encoded toxin was strongly linked to the lineages CC30 (40/48) and CC395 (5/5), and only rarely detected in other *S. aureus* CCs if at all (**Figure 1** and **Supplementary Table 2**).

**Figure 2:**
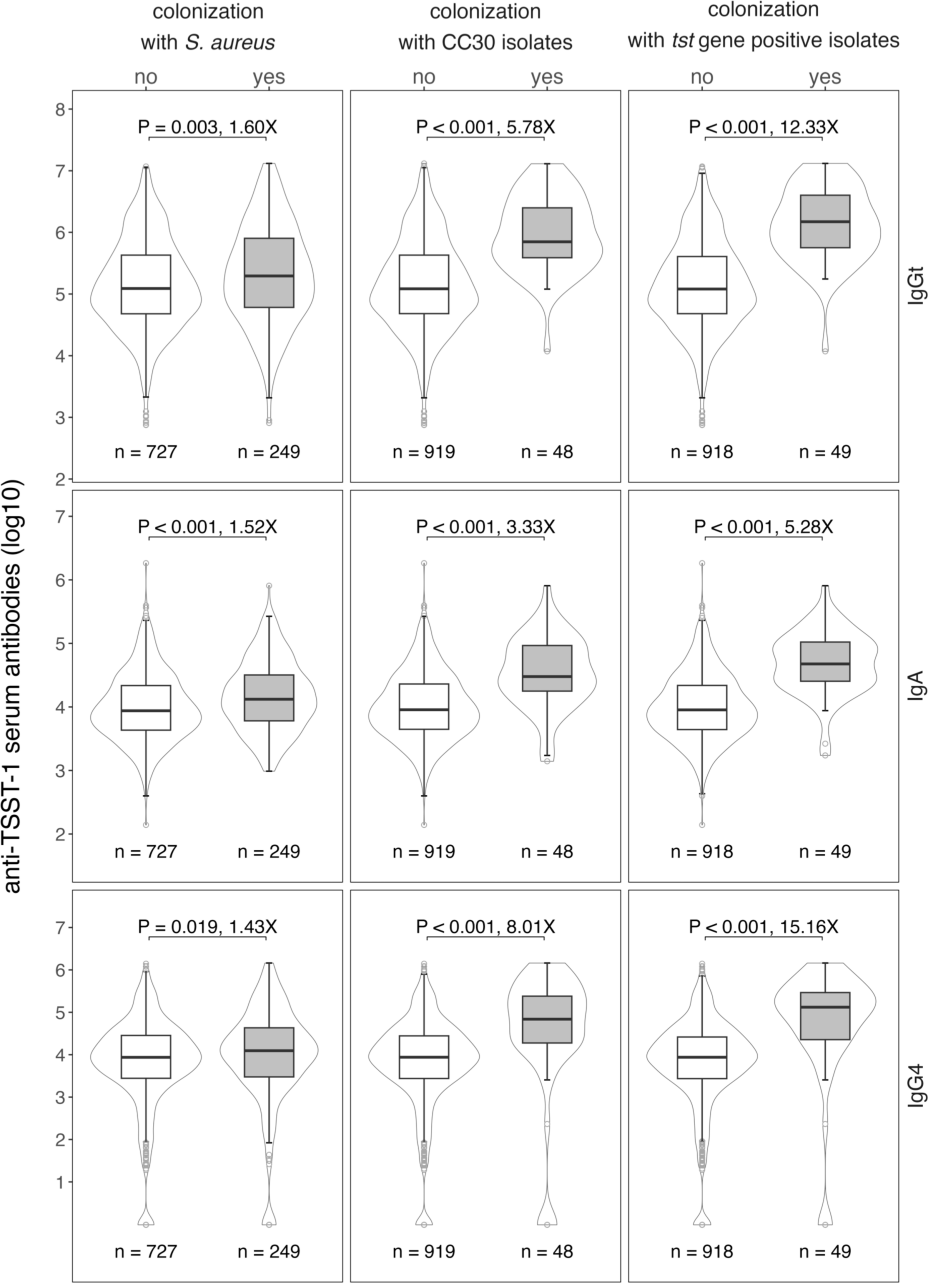
High anti-TSST-1 antibody levels in the general population are linked to *S. aureus* colonization, in particular colonization with *tst*-positive *S. aureus* strains. Anti-TSST-1 IgGt (top panel), IgA (middle panel) and IgG4 (bottom panel) antibody levels were quantified using the xMAP^®^ technology (19, 32). Data were stratified by *S. aureus* nasal colonization (left panel), colonization with CC30 *S. aureus* strains (central panel), or colonization with *tst*-positive *S. aureus* strains (right panel). CCs were determined by *spa* typing, the presence of *tst* was detected by multiplex PCR (11, 33). Box plots display the median along with the 25th and 75th quantiles, whiskers depict the 25th quantile minus 1.5*IQR and 75th quantile plus 1.5*IQR, respectively. Probability density shown as violin plots. Statistics: unpaired Wilcoxon rank sum test; X, fold-change between median values.

To investigate whether exposure to TSST-1 affects the levels of anti-TSST-1 antibodies, we quantified TSST-1-binding antibody levels and correlated them with current *S. aureus* nasal carriage. Anti-TSST-1 total IgG (IgGt), IgA, and IgG4 antibodies were measured in serially diluted serum samples over a broad detection range using the xMAP^®^ technology. TSST-1-specific serum IgGt levels varied over a factor of 4.2 logs with no major difference between males and females or age groups (**Figure 2** and **Supplementary Figure 1**). *S. aureus* carriers exhibited 1.6-fold higher specific IgGt levels than non-carriers (**Figure 2**, top panel), suggesting that recent exposure increases antibody titers. A broad range of anti-TSST-1 antibodies in non-carriers, however, implies that these individuals have also been exposed to the TSST-1 toxin in the past. As the presence of the gene *tst* is strongly linked with CC30, we next stratified our analysis by this most common *S. aureus* lineage. As expected, the difference in antibody titers was much more pronounced when comparing individuals carrying CC30 strains with non-carriers and individuals colonized with other *S. aureus* CCs (5.8-fold). Finally, individuals carrying a *tst*-positive *S. aureus* strain had 12.3 times higher serum IgGt binding to TSST-1 than those who did not (non-carriers and carriers of *tst*-negative strains) (**Figure 2**, top panel).

The anti-TSST-1 IgA response resembled the IgGt response in many aspects, but IgA levels were generally lower than IgGt levels (**Figure 2**, middle panel). Again, we observed a broad range of TSST-1 serum IgA antibody titers covering 4.1 logs, IgA levels were by a factor of 5.3 higher in carriers of *tst*-positive *S. aureus* strains as compared to non-carriers and carriers of *tst*-negative strains. We also assessed the IgG4 antibody response as a marker for type 2-driven immune responses. Thirty-seven individuals (3.8%) lacked TSST-1-specific IgG4 and 15.2% (148/976) of the cohort presented with antibody levels below 1,000 AU. Nevertheless, carriage of CC30- or *tst*-positive strains again strongly affected the anti-TSST-1 IgG4 levels (**Figure 2**, bottom panel).

**Figure 3:**
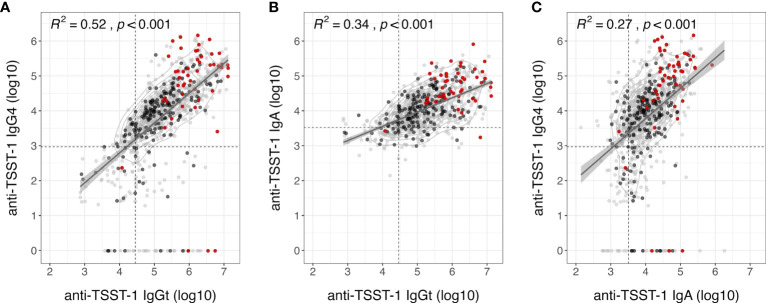
Moderate correlation of anti-TSST-1 IgGt, IgA and IgG4 antibody levels. Correlation plots of anti-TSST-1 IgGt vs. IgA **(A)**, IgGt vs. IgG4 **(B)**, and IgA vs. lgG4 **(C)**. Colors indicate *S. aureus* non-carriers (gray), carriers colonized with *tst*-negative strains (black), and carriers colonized with *tst*-positive strains (red). Data points are semi-transparent to better visualize areas with high point densities. We performed a 2D kernel density estimation and displayed results as contours. Dashed lines indicate the 15^th^ percentiles in antibody levels, which could indicate a threshold of protection (IgGt: 28,320 AU, IgA: 3,211 AU, IgG4: 931 AU) (27, 28). Diagonal lines with 95% confidence intervals (shaded gray areas) indicate a linear fit based on a simple linear regression model. R², square of the spearman correlation coefficient rho; p, p-value of spearman correlation.

There was a fair correlation between IgGt and IgG4 (R² = 0.52) – although some individuals with high IgGt titers lacked IgG4 completely – and between IgGt and IgA (R² = 0.35), as well as a poor correlation between IgA and IgG4 (R² = 0.27) (**Figure 3**) (34). Previous studies estimated that up to 15% of the population lack protective titers of TSST-1 neutralizing serum antibodies (27, 28). As TSST-1-binding and -neutralizing antibodies correlate well (26), we applied this 15% cut-off to our IgGt, IgA, and IgG4 data sets. Only one out of the 49 carriers of *tst*-positive strains had an IgGt titer below this 15^th^ percentile. Similarly, only 2 out of 49 carriers of *tst*-positive strains showed anti-TSST-1 IgA below this threshold. In summary, anti-TSST-1 antibody levels were highly variable. Recent TSST-1 exposure due to nasal colonization with *tst*-positive *S. aureus* strains strongly increased the anti-TSST-1 antibody titers.

### 2.2 Anti-TSST-1 antibody levels are associated with SNVs in the HLA class II region

**Figure 4:**
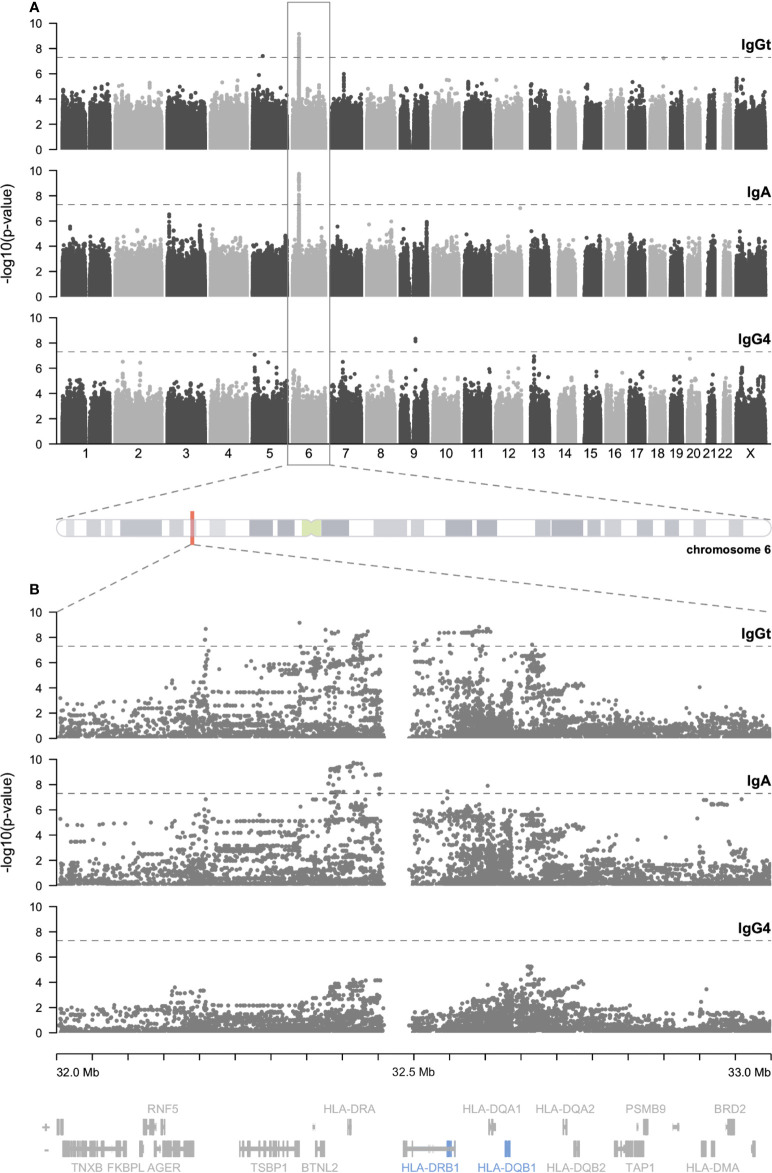
Manhattan plots demonstrate genome-wide associations of anti-TSST-1 IgGt and IgA antibody levels with HLA-DQ and DR loci. SNVs were plotted on the x-axis according to their position on each chromosome against the association with the respective phenotype (anti-TSST-1 IgGt, IgA, and IgG4 levels) on the y-axis (shown as -log_10_ p-value) **(A)**. The GWAS are based on 969 SHIP-TREND-0 participants. The dashed line indicates the threshold for genome-wide significance (p=5x10^-8^). Regional plots of the genomic region on chromosome 6 covering the significant SNVs (32 – 33 Mb) **(B)**.

To identify genetic determinants of the anti-TSST-1 antibody response, we performed genome-wide association studies (GWAS) on the phenotypes anti-TSST-1 IgGt-, IgG4-, and IgA levels. We included all SHIP-TREND-0 participants of our study sample with available antibody profiles and genome-wide SNV typing information (n=969). The latter was generated by individual array-based typing using genomic DNA prepared from whole blood and the Illumina Infinium Omni2.5 BeadChip as described before (30, 31). Additional SNV alleles were imputed using the HRC (r1.1 2016) imputed dataset (35). We observed a strong association of the anti-TSST-1 IgGt and IgA levels with the HLA class II genes DQ and DR on chromosome 6 (**Figure 4A**). In contrast, the anti-TSST-1 IgG4 levels were not associated with the HLA class II complex, and only weakly with other loci.

The identified region on chromosome 6 encodes the α and β chains of the MHC class II molecules HLA-DQ, and -DR, which are involved in antigen presentation to helper T cells (**Figure 4B**). The β chains of both MHC molecules are highly polymorphic with several thousand reported allelic variants (HLA- DQB1: 2419 alleles, DRB1: 3486 alleles), while the α chains are less variable (DQA1: 556 alleles, DRA: 46 alleles) (https://www.ebi.ac.uk/ipd/imgt/hla/about/statistics/. IPD-IMGT/HLA Database. Version report - 3.51). To reliably identify the involved HLA-DQB1 and -DRB1 alleles, we HLA-typed sub-groups of our cohort by either targeted sequencing of the β chains of the HLA class II gene loci or whole genome sequencing (WGS).

### 2.3 HLA-DQ and -DR alleles are associated with anti-TSST-1 antibody levels

**Figure 5:**
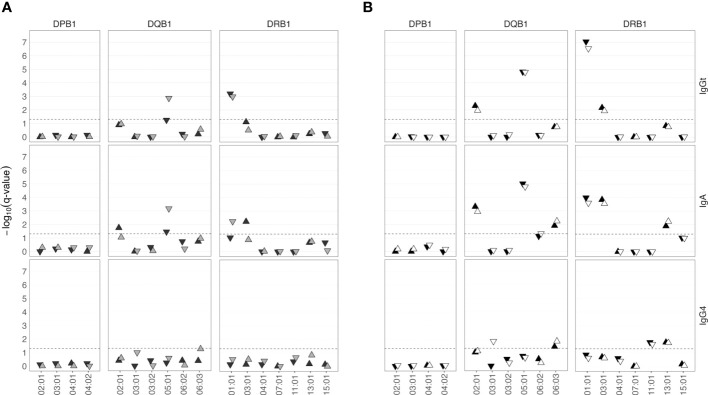
HLA class II alleles are associated with low or high anti-TSST-1 IgGt and IgA antibody levels. **(A)** A sub-cohort of 574 SHIP-TREND-0 participants was HLA-typed by PCR amplification and sequencing of the HLA coding regions (DKMS, light gray). The most frequent HLA class II alleles (frequency ≥ 3%) were correlated with the anti-TSST-1 antibody titers. The findings were validated by deducing HLA types from Illumina whole genome sequencing data using the HLA-HD algorithm (n=402, dark gray). **(B)** Meta-analysis of the results from both approaches (n=976, black). DNA array-based HLA allele prediction using the Four-digit Multi-ethnic HLA v2 imputation panel (n=965, white). –log_10_(q-value), negative decadic logarithm of the Benjamini-Hochberg (BH) adjusted p-value.

First, we determined the β chains of the HLA class II alleles as well as the HLA class I alleles in a sub-group of 574 SHIP-TREND-0 participants by PCR amplification and subsequent sequencing of the respective genomic regions. Sequencing was performed by the German Bone Marrow Donor Center (Deutsche Knochenmarkspenderdatei, DKMS), which coordinates the largest HLA-typed donor registry in Germany (36). The most frequent HLA class II alleles (frequency ≥ 3%) were correlated with anti-TSST-1 antibody binding. In this screening sample, HLA-DQB1*05:01 and HLA-DRB1*01:01 were negatively associated with the strength of the serum IgGt binding to TSST-1 (**Figure 5A**). The same alleles were negatively associated with TSST-1-binding IgA antibodies. In contrast, anti-TSST-1 IgG4 levels were not associated with HLA class II alleles. In line with the GWAS results, we observed no correlation of the HLA-DP locus and the HLA class I loci (A, B, and C) with antibody binding to TSST-1 (**Figure 5A** and **Supplementary Figure 2**).

To validate our findings, we determined the HLA type in the so far untyped SHIP-TREND-0 participants using available WGS data (n=402). Both HLA class I loci and class II loci (including also the α chains) were derived from Illumina WGS data using the HLA-HD algorithm (37). WGS data confirmed the association of anti-TSST-1 IgGt and IgA with HLA-DRB1*01:01, and found a similar trend for HLA-DQB1*05:01 (**Figure 5A**). In addition, HLA-DQB1*02:01 and HLA-DRB1*03:01 were positively associated with anti-TSST-1 IgA titers.

To increase the statistical power, we next combined the HLA typing data from the two groups, HLA typed by DKMS or WGS, in a meta-analysis based on the summary statistics from the single variant association analyses (**Figure 5B**) (38). This approach confirmed the results and identified additional associations of HLA alleles with anti-TSST-1 antibody levels. Again, HLA-DQB1*05:01 and DRB1*01:01 were negatively associated with the strength of the serum IgGt reaction to TSST-1. In addition, HLA-DQB1*02:01 and HLA-DRB1*03:01 now also exhibited a significant positive association with TSST-1-binding IgGt antibodies. The very same alleles were positively or negatively associated with TSST-1-binding IgA antibodies. In addition, the alleles HLA-DQB1*06:03 and HLA-DRB1*13:01 were positively associated with IgA levels and showed a similar trend for IgGt. In contrast, anti-TSST-1 IgG4 levels were associated with a different set of HLA-DQ and -DR alleles, albeit weakly.

HLA sequencing data or WGS data are rarely available for population-based cohort studies similar to SHIP. Hence, we explored whether the HLA type can be reliably deduced from array-based genotyping data for all individuals with available genotyping data and anti-TSST-1 antibody profiles (n=965). We determined the HLA type, covering both α and β chains for HLA class II, by applying the recently published Four-digit Multi-ethnic HLA v2 (2022) algorithm (39) to genotyping data that had been obtained with the Illumina Infinium HumanOmni2.5 BeadChip. Again, we retrieved exactly the same panel of positively and negatively associated HLA-DQB1 and -DRB1 alleles. We also observed an association of anti TSST-1 IgGt and -IgA with DQA1 alleles (**Supplementary Figure 3**). The log10 p-values were highly concordant between the array-based and the sequencing-based HLA data (**Figure 5B**). A comparison of the imputed HLA alleles with the sequence-based HLA typing (DKMS) confirmed 98.97% and 98.67% accuracy in HLA class I and II allele prediction based on the four-digit code. Hence, reliable prediction of HLA alleles from genotyping data is feasible, making HLA analysis easily available to numerous population-based studies.

To summarize, both a sequencing-based meta-analysis and an array-based genotyping approach revealed that HLA-DQB1*02:01 and HLA-DRB1*03:01 were positively, and HLA-DQB1*05:01 and HLA-DRB1*01:01 negatively associated with anti-TSST-1 IgGt and IgA serum levels.

**Figure 6:**
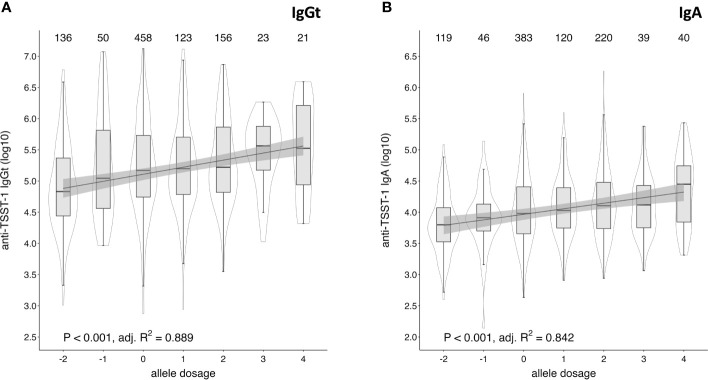
Cumulative effect of positively and negatively associated HLA alleles on anti-TSST-1 IgGt and IgA levels. Allele dosage effects of positively and negatively associated HLA-DQB1 and -DRB1 alleles on anti-TSST-1 IgGt **(A)** and IgA **(B)** were assessed by assigning positively associated alleles the arbitrary value of 1 (IgGt: DQB1*02:01, DRB1*03:01; IgA: additionally, DQB1*06:03 and DRB1*13:01) and negatively associated alleles the value of -1 (IgGt and IgA: DQB1*05:01, DRB1*01:01). The analysed subsample comprised all DKMS- and WGS-based HLA typed individuals (n=976). The allele dosage was defined as the sum of those values. Only allele dosage groups with case numbers ≥ 20 were included in the correlation analysis. Box plots display the median along with the 25th and 75th quantiles, whiskers depict the 25th quantile minus 1.5*IQR and 75th quantile plus 1.5*IQR, respectively. Probability density shown as violin plots. Statistics: p-value and adjusted R squared by simple linear regression model of median log_10_-transformed anti-TSST-1 antibody levels and allele dosage categories.

Due to the high polymorphism of the HLA locus, most individuals are heterozygous for the genes encoding the HLA type I and II molecules. Since HLA alleles are co-dominantly expressed, most individuals will express two HLA-DR and -DQ alleles and thus could potentially harbor combinations of up to four positively and/or negatively associated alleles. To estimate a possible dosage effect, we assigned each positively and negatively associated allele with an arbitrary value of 1 and -1, respectively, and calculated the sum of all alleles that were significantly associated with TSST-1-specific IgGt and IgA responses for each individual (**Supplementary Table 3**). The obtained dosage score correlated with the IgGt and IgA responses to TSST-1, showing increasing median antibody levels with increasing dosage score (**Figure 6** and **Supplementary Figure 4**). We focused on dosage groups with ≥ 20 individuals (dosage groups -2 to 4) and conducted a regression analyses based on the median antibody levels. This confirmed a cumulative effect of the number of positively or negatively associated HLA alleles on the strength of the anti-TSST-1 IgGt and IgA response. The maximal effect size was a 4.9-fold increase in median antibody levels between the dosage groups -2 and 4 for IgGt and a 4.5-fold increase for IgA. Since HLA-DQ and -DR were only moderately correlated with IgG4 binding to TSST-1, there was no clear dosage effect for this antibody class (data not shown).

If we assume that 15% of the population are at an increased risk of TSS because of low toxin-specific antibody levels, 25.6% (50/195) of those with predominantly negative HLA alleles but only 8.7% (28/323) of those with predominantly positive HLA alleles belong to this vulnerable group. In summary, we demonstrated a cumulative effect of positively and negatively associated HLA alleles on anti-TSST-1 IgGt and IgA levels.

## 3 Discussion

Most adults have protective titers of anti-TSST-1 antibodies, but up to 15% of the population is considered at risk for TSS. We have identified determinants of the anti-TSST-1 antibody response in the population-based study SHIP-TREND-0. Anti-TSST-1 IgGt antibody levels were highly variable, with 4.2 orders of magnitude difference between low and high responders. Recent exposure by nasal colonization with *tst*-positive *S. aureus* strains enhanced antibody levels by a factor of 12.3. In addition, we observed a strong genetic correlation with HLA class II alleles. HLA-DRB1*03:01 and HLA-DQB1*02:01 were positively, and HLA-DRB1*01:01 and HLA-DQB1*05:01 negatively associated with anti-TSST-1 antibody levels. Antibody levels differed by a factor of 4.9 between individuals harboring negatively-associated HLA-DR and -DQ alleles and those with 4 positively associated alleles. Hence, both toxin exposure and HLA alleles have a strong impact on the antibody response to TSST-1.

Using a sensitive Luminex^®^ assay, we were able to measure anti-TSST-1 serum antibodies in every study participant, with highly variable titers between individuals. In contrast, previous studies employing ELISA or RIA failed to detect anti-TSST-1 antibodies in approximately 8% of the young general population (<30 years) (27, 40). The higher sensitivity of our analyses is a feature of the Luminex^®^ system. By measuring each serum in seven dilutions, we achieved a dynamic range of 5 orders of magnitude. We calculated the relative specific antibody levels (AU) with the new data analysis tool xMAPr (32). In this study, we determined only TSST-1-binding, however, binding and neutralizing antibody titers against TSST-1 are strongly correlated. In detail, we observed a strict correlation between antibody binding as determined by Luminex^®^ assay and TSST-1 neutralizing capacity (inhibition of TSST-1-induced T cell proliferation) in a cohort of healthy *S. aureus* carriers and non-carriers (26). Mouse studies corroborate this and show that SAg binding (SEA, SEB, SEC, TSST-1, as determined by ELISA) strictly correlates with protection (41).

TSST-1-neutralizing antibodies protect against TSS, while low antibody titers put individuals at risk (27, 29, 42). There is anecdotal evidence that the application of intravenous Ig preparations, which are prepared from large human plasma pools and contain TSST-1-neutralizing antibodies, can decrease mortality in staphylococcal TSS (42, 43). ELISA-based studies from the 1980s suggest that serum antibody titers below 100 will not provide sufficient protection (27, 29, 44). These studies estimated that up to 15% of the general adult population lack sufficient antibody titers. Assuming a similar seroprevalence, this would mean that up to 146 individuals in our study sample were at an increased risk of TSS. This corresponds to a cut-off of 28,320 AU for anti-TSST-1 IgGt, which is 10 times lower than the median IgGt level.

Anti-TSST-1 antibodies are generated by direct exposure to the toxin during colonization or infection (20–22). The most frequent route of TSST-1 exposure is nasal (and vaginal) colonization. In our study, 5.02% of the participants were intranasally colonized with *tst*-positive strains, which is comparable to data from North America (40). Vaginal colonization with TSST-1-producing *S. aureus* reportedly ranged between 1-3% (40, 45, 46). Moreover, TSST-1-producing *S. aureus* was isolated from 4% of tampons retrieved from healthy menstruating women (13). The relatively low frequency of colonizing *tst*-encoding *S. aureus* strains as compared to 100% seroconversion to TSST-1 suggests frequent fluctuation of *S. aureus* strains in the general population. Nevertheless, in some adults the immune system was probably never directly confronted with TSST-1. Antibody binding, especially at low levels, could be due to cross reactivity with other SAgs, despite the limited sequence similarity (33-39%; data not shown) (22, 41, 47, 48). Moreover, environmental and behavioral factors could influence seroconversion (28). Thus, individuals with very low or lacking anti-TSST-1 antibodies have either never encountered TSST-1 or were inherently incapable of developing a TSST-1 antibody response. This is consistent with the results of the first clinical TSST-1 vaccine trial. 46 individuals were immunized (with adjuvant) with a recombinant TSST-1 toxoid (dose range: 100 ng - 30 µg). Only two vaccinees failed to produce specific antibodies; both had received a very low antigen dose (100 ng or 300 ng) (49, 50). This illustrates that upon exposure with significant amounts of TSST-1, almost everyone generates antibodies.

Recent exposure by nasal *S. aureus* colonization strongly enhanced anti-TSST-1 antibody titers. On average, antibody levels were 12.3-fold higher in carriers of *tst*-positive *S. aureus* strains than in the rest of the cohort comprising noncarriers and carriers of *tst*-negative strains. In an earlier study, 98% of menstruating women colonized with *tst*-positive strains had measurable anti-TSST-1 antibodies as compared to 84% in noncarriers or carriers of *tst*-negative strains (40). In line with this, our group previously reported that *S. aureus* carriers mount a strong antibody response against the SAgs produced by their colonizing strain (17). This suggests that colonization with a *tst*-positive *S. aureus* strain, either *per se* or due to minor subclinical infections, induces an anti-toxin antibody response. Among the 49 carriers of *tst*-positive *S. aureus* strains, only one had IgGt and two had IgA levels below the 15% percentile, placing them at increased risk of developing TSS. This implies that given sufficient exposure to TSST-1 almost all adults can generate specific antibodies against the toxin. Whether the antibodies induced by intermittent colonization are stable over time, remains to be clarified as longitudinal data on anti-*S. aureus* antibodies are not available in the SHIP-TREND cohort. Such data are generally scarce (26). The determination of the stability of anti-*S. aureus* toxin antibodies is an important topic to be addressed in future studies, since there are examples for both extremely stable humoral immune memory in humans and a rapid decline of antibody titers after cessation of exposure (51, 52).

Our GWAS revealed a robust association of anti-TSST-1 IgGt and IgA levels with HLA-class II but not with HLA-class I alleles. HLA typing demonstrated that HLA-DRB1*03:01 and HLA-DQB1*02:01 were positively, and HLA-DRB1*01:01 and HLA-DQB1*05:01 negatively associated with the anti-TSST-1 IgGt serum levels. The effect size was 4.9-fold when comparing individuals harboring all four positively associated alleles and those with a cumulative allele dosage of -2. Hence, HLA alleles have a smaller impact than recent TSST-1 exposure. If we assume that 15% of the adult population has antibody levels too low to protect against TSS, 25.6% (50/195) of those with predominantly negative HLA alleles versus 8.7% (28/323) of those with predominantly positive HLA alleles belong to this vulnerable group. Whether the reported negatively-associated alleles are enriched in TSS patients will be in the focus of future projects. The selective association of HLA class II with the TSST-1-specific antibody response can be explained by their behavior as a conventional protein antigen. HLA-alleles that present a broader spectrum of TSST-1 peptide epitopes could potentially activate more T helper cells resulting in more effective help to TSST-1-specific B cells and higher anti-TSST-1 antibody titers.

The determinants of the IgG4 response to TSST-1 differed from those influencing the IgGt and IgA response. While the TSST-1-specific IgG4 titers were also associated with colonization by *tst*-positive *S. aureus* strains, some individuals had no measurable toxin-binding IgG4 at all. Second, serum IgG4 was only weakly affected by the HLA-II polymorphism. The distinct behavior of the IgG4 response to TSST-1 is not surprising, as function and regulation of IgG4 are special (53). IgG4 is not usually associated with antimicrobial defense but rather with immune responses to worms and with allergies. There is further a broad spectrum of rare IgG4-related diseases characterized by inflammatory dysregulation (53–55). IgG4 acts mainly by competing with other Ig classes for epitope binding and has little inflammatory effect itself (53). IgG4 release is regulated similarly to IgE. Both Ig subclasses are generated by B cells under the influence of IL-4 and IL-13, which requires the help of Th2 cells, more specifically follicular T helper cells type 2 (56). Since IgG4 production additionally requires IL-10 derived, for example, from regulatory T cells, this is referred to as a modified Th2 response (57–59). Further, HLA alleles play a role in IgG4 production. There is a strong, cross-disease association with HLA-DQB1*05 (60). This HLA allele apparently predisposes to IgG4 generation, but, as shown in this study, it antagonizes the humoral immune response to anti-TSST-1. We conclude that two independent conditions must be met for the development of TSST-1-specific IgG4 (i): exposure to TSST-1 and (ii) a genetic predisposition favoring a modified type 2 response and a class switch to IgG4.

To which extent variations in total antibody levels in the blood may have biased the TSST-1-specific antibody titers could not be measured in this study, because data on total IgG, IgA, and IgG4 subclasses are not available in the SHIP-TREND-0 cohort. However, variations in total IgG and IgA serum levels are small (reference ranges IgG: 7,0 – 16.0 g/L; IgA: 0.7 – 4.0 g/L) in comparison with the large differences in anti-TSST-1 IgGt and IgA antibody levels, spanning 4-5 orders of magnitude and therefore cannot explain them (61, 62). Total IgG4 serum concentrations, however, are more variable (reference range: 0.004–2.68 g/L). In line with this, many individuals in our cohort had no measurable or very low TSST-1-specific IgG4 titers. Thus, in the case of IgG4, factors other than antigen exposure and HLA class II polymorphism also have an important influence.

Besides influencing anti-TSST-1 antibody titers, HLA class II polymorphisms can also directly impact on TSST-1 binding and thus SAg-driven pathogenesis. Of note, SAg-mediated cross-linking of MHC class II and TCR is not occurring simultaneously. It is initiated by high affinity binding of these toxins to HLA class II molecules on the APC surface, followed successive engagement and cross-linking of multiple TCR molecules (63). Several *in vitro* studies demonstrated that the binding of SAgs to HLA class II is influenced by HLA class II loci and polymorphism. TSST-1 binds effectively to HLA-DR, but not HLA-DP (64, 65). In addition, HLA class II alleles differ strongly in their capacity to induce T cell proliferation and cytokine secretion upon SAg stimulation (66). Interestingly, there are also qualitative differences in the SAg-induced T cell response, as reflected by different cytokine patterns and modified TCR Vβ profiles (66). The influence of HLA-DR polymorphisms on the immune response to TSST-1 could be confirmed in HLA transgenic mice (67). Moreover, HLA class II haplotypes influence the outcome of invasive group A streptococcal infections (68). HLA class II alleles that were associated with protection from severe systemic manifestations of disease presented streptococcal SAgs in a manner that resulted in significantly reduced cytokine and proliferative responses (68). On top, TSST-1-induced T cell activation is also affected by the bound peptide antigen, because this toxin forms a wedge between HLA class II and TCR and directly contacts the bound peptide antigen (69, 70). Together, the overall avidity of the HLA:peptide - TCR - TSST-1 complex likely determines the strength and also quality of the TSST-1-driven immune response.

The HLA-alleles are strongly associated with numerous diseases. However, in many population-based studies, HLA-haplotypes have not been determined due to technical difficulties in resolving the extreme polymorphism of the HLA gene locus. Our study shows how this gap may be closed by imputation in many cases.

The classical HLA class I and class II molecules mediate the antigen-specific activation of T cells, which in turn orchestrate the adaptive immune responses. Since the locus is highly polymorphic and HLA alleles present distinct spectra of antigenic peptide epitopes to T cells, the HLA haplotypes impact on a variety of diseases with large effect sizes, in particular, autoimmune disorders, immune deficiencies, inflammatory diseases, and infectious diseases (71). Nevertheless, in GWASs, the HLA gene locus is often excluded because it is one of the most challenging genome regions to study due to its extreme polymorphism, its complex relationship with natural selection, and its unique long-range linkage disequilibrium structure (72). On top, the genetic diversity at the HLA gene locus is highly population-specific, necessitating balanced global reference panels. PCR-based methods are still the gold standard in HLA typing (36), but recently developed algorithms, such as “HLA-HD”, now also enable high- to full-resolution HLA genotyping based on NGS data (37, 73, 74).

Since to date HLA sequencing data or NGS data are often not available in population-based studies, we explored whether the HLA type can also be reliably imputed *in silico* from array-based genotyping data. Therefore, in addition to the HLA typing by direct sequencing, we imputed the genotype information (based on Illumina Infinium HumanOmni2.5 BeadChip genotyping data) on the Michigan Imputation server (MIS) to the four-digit Multi-ethnic HLA v2 (2022) panel for the set of 976 samples (39, 72). A comparison of the imputed HLA alleles with the sequence-based HLA typing (DKMS) confirmed 98.97% and 98.67% accuracy in HLA class I and II allele prediction, respectively, based on the four-digit code. In line with our data, Luo et al. reported 97.8% accurate imputation at G-group resolution in European populations using the same reference panel (39). Thus, reliable prediction of HLA alleles from genotyping data is feasible and as accurate as NGS-based HLA typing. This means that now HLA alleles can be reliably determined in the millions of individuals that have been genotyped on microarrays, promising new insights into the genetics of infectious diseases, autoimmune disorders, immune deficiencies, and inflammatory disorders.

Our study population is generally very homogeneous, which is a limitation of the work we present in this manuscript. Other populations could differ (i) in molecular epidemiology of endemic *S. aureus*, (ii) HLA-haplotype distribution and (iii) numerous other genes and environmental factors that influence the immune response. Variability of HLA alleles across different ethnicities is well-documented (75). The outcome of the analysis could therefore differ between groups of different ancestry. In particular, there may be additional associations between HLA and TSST-1 antibodies that were undetectable in our cohort because of low allele frequency. A Japanese study reported a similar prevalence of TSST-1 producing *S. aureus* in Japan and the US, while antibody levels in the Japanese cohort were much lower. Notably, both genetic and environmental factors appeared to be important in promoting the development of anti-TSST-1 antibodies, as antibody titers in Japanese women living in Tokyo, were higher than in those living in the United States, but still lower than titers in Caucasian US women (28).

Our research has implications for *S. aureus* vaccine development. We found that only very few individuals may be inherently incapable of developing a TSST-1 antibody response. Nevertheless, a substantial fraction of the adult population has low specific antibody titers and would benefit from an antibody boost induced by a TSST-1 vaccine. This would not only protect them from TSS, but also from other staphylococcal diseases with SAg involvement, e.g., sepsis, and osteomyelitis.

## 4 Methods

### 4.1 SHIP-TREND-0

The Study of Health in Pomerania (SHIP) is a longitudinal population-based study which aims to determine the incidence and prevalence of common risk factors, subclinical disorders and clinical disease (30, 31). The SHIP-TREND cohort only includes individuals of European ancestry. The detailed study design has been published previously (30, 31). SHIP-TREND-0 is the baseline assessment of the 2^nd^ SHIP cohort with proband recruitment (N = 4,420) conducted in the Northeast of Germany between 2008 and 2012. In depth OMICs data (whole genome sequencing, whole-blood-transcriptome and methylome, plasma circulating miRNome, plasma proteome, plasma, urine and saliva metabolome as well as stool and saliva microbiome) as well as *S. aureus* antibody profiles, however, are only available for a subcohort of 996 subjects (19, 30). Further 20 individuals had to be excluded from our analyses because information on the colonizing *S. aureus* strain were not available (n=11), HLA typing via DMKS was unsuccessful and WGS not available (n=7), or smoking status was not available (n=2). The study protocol of SHIP-TREND-0 was approved by the local ethics committee of the University of Greifswald (registration no. BB39/08) with all participants giving informed written consent.

### 4.2 Nasal *S. aureus* colonization

Nasal *S. aureus* colonization was determined by swabbing both nasal vestibules with a rayon swab (BBL CultureSwab Liquid Stuart; BD, USA) and subsequent cultivation of retrieved bacteria in phenol red mannitol salt broth and on mannitol salt agar (BD, Heidelberg, Germany) as described in detail elsewhere (11).

### 4.3 *S. aureus* genotyping and *tst* gene detection

*spa* genotyping of *S. aureus* strains was performed according to published protocols using the primers spa-1113f and spa-1514r (76). Closely related *spa* types (costs ≤ 3) were grouped into *spa* clonal complexes (*spa* CCs) using the BURP algorithm (77). *Spa* CCs were subsequently assigned to multilocus sequence type (MLST) CCs using the SpaServer database (www.spaserver.ridom.de). The gene *tst* gene was detected by multiplex-PCR as previously reported (33).

### 4.4 Anti-TSST-1 IgGt, IgA, and IgG4 antibody levels

Anti-TSST-1 (locus ID: SA1819) total IgG (IgGt), and IgA antibodies were quantified using a bead-based suspension array (xMAP^®^ technology, Luminex^®^) as described in detail elsewhere (19, 32). TSST-1-specific IgG4 was quantified with an R-Phycoerythrin-labelled anti-human IgG4 (Southern Biotech, order number 9200-09). An arbitrary antibody binding value (AU) was calculated from the dilution series based on curve fitting algorithms using the xMAPr analysis tool (32). If curve fitting failed, an imputation was performed based on a LOESS fit over all measurements of a single dilution (19).

### 4.5 HLA typing (DKMS)

HLA typing of 574 SHIP-TREND-0 samples for *HLA-A, B, C, DPB1, DQB1*, and *DRB1* was performed at the DKMS (Deutsche Knochenmarkspenderdatei) Life Science Lab Dresden (36). The respective genomic regions were amplified by PCR and subsequently sequenced. Allele codes were allocated by either G groups or Multiple Allele Code (MAC) using IPD-IMGT/HLA database version 3.36.0 (https://www.ebi.ac.uk/ipd/imgt/hla/release/v336/).

### 4.6 HLA typing (NGS)

HLA typing based on WGS results for 408 SHIP-TREND samples was performed using HLA-HD version 1.5.0 with default parameters (37, 78). The software deduces *HLA-A, B, C, DPA1, DPB1, DQA1, DQB1, DRA*, and *DRB1* alleles along with some non-classical HLA genes. HLA-HD dictionary was updated to IPD-IMGT/HLA database version 3.36.0 to match the database used by the DKMS. Typing was done on paired-end whole genome sequencing fastq data. HLA-HD allocated the alleles predominately in 4- or 6-digit field codes.

### 4.7 HLA imputation (genotyping array)

Genotyping of 983 SHIP-TREND-0 sample was initially performed using the Illumina Infinium HumanOmni2.5 BeadChip. Pre-imputation data generation and quality control of genotyping results was conducted according to the guidelines provided by the imputation service. Genotype information was imputed on the Michigan Imputation server (MIS) to the four-digit Multi-ethnic HLA v2 (2022) panel which is based on IPD-IMGT/HLA database version 3.32.0 (https://www.ebi.ac.uk/ipd/imgt/hla/release/v332/) (39). This panel provides information on *HLA-A, B, C, DPA1, DPB1, DQA1, DQB1*, and *DRB1* alleles.

### 4.8 Harmonization of HLA alleles

DKMS-allocated MAC designations were converted to G groups using the MAC Service 2.12.1-SNAPSHOT UI provided by the National Marrow Donor Program (https://hml.nmdp.org/MacUI/). All allele codes derived by the abovementioned methods were restricted to a two-field code (four-digit) and assigned to the associated G groups.

### 4.9 HLA allele association analyses

For each HLA typing method (WGS-based, DKMS, and imputation-based) we conducted linear regression analyses and implemented an additive genetic model to evaluate the association of log10-transformed anti-TSST-1 antibody levels on the HLA allele occurrence (0, 1, or 2 copies of the allele). Only individuals with complete data on HLA typing, and anti-TSST-1 antibody levels were included. We adjusted for the covariates age (years), sex, body-mass index (BMI, kg/m^2^), *S. aureus* colonization status, and smoking status (current, former and non-smoker). These confounders were identified in a previous study investigating anti-*S. aureus* serum IgGt levels against a panel of 79 *S. aureus* antigens (19).

### 4.10 Meta-analysis

We used the R package metaphor (v3.8.1) to perform a meta-analysis on the summary statistics from the single variant association analyses for WGS- and DKMS-based HLA typing (38). We applied the implemented fixed-effects model with weighted estimation.

### 4.11 Cumulative effect of HLA-DQ and -DR genes

A dosage effect was determined by assigning protective HLA-DQB1 and -DRB1 alleles (as determined by the abovementioned HLA typing approaches) with an arbitrary value of 1 and risk alleles with a value of -1 and summing up the protective and risk alleles on both chromosomes for each individual.

### 4.12 Data visualization and statistical analysis

All statistical testing and data visualization was performed in R (v4.2.0; https://www.R-project.org/) in combination with the tidyverse package (v2.0.0) (79), rstatix (v0.7.2; https://CRAN.R-project.org/package=rstatix), metaphor (v3.8.1) (80), and plotgardener (v1.4.2) (81).

## Data availability statement

The data analyzed in this study is subject to the following licenses/restrictions: The data protection policy of the Study of Health in Pomerania (SHIP) does not permit open access to SHIP data or transfer to journals. Data can be accessed for reuse and quality control/assurance via SHIP’s standardized data usage application procedure. Requests to access these datasets should be directed to https://transfer.ship-med.uni-greifswald.de. Summary statistics of all data are provided in **Supplementary Figure 4**.

## Ethics statement

The studies involving human participants were reviewed and approved by the Clinical Ethics committee of the University Medicine Greifswald, Greifswald, Germany. The patients/participants provided their written informed consent to participate in this study.

## Author contributions

Conceptualization, UV, SH, and BB. Methodology, SW. Software: SW. Formal Analysis, SW, SM. Investigation, SW, TM, CM, CC. Resources, MD, SF, HG, GH, CK, MN, NF, SS, HV, UV, BB. Data Curation, SW. Writing – Original Draft, FS, SH, SW. Writing – Review & Editing, all authors. Visualization, SW, SM. Supervision, UV, SH, and BB. Funding Acquisition, UV, BB, SF, MD, NF, GH, MN, HG. All authors contributed to the article and approved the submitted version.
