# Supplementary material for: Toxin exposure and HLA alleles determine serum antibody binding to toxic shock syndrome toxin 1 (TSST-1) of Staphylococcus aureus
Source: Front Immunol. 2023 Sep 4;14:1229562. doi: 10.3389/fimmu.2023.1229562 (PMC10507260; doi:10.3389/fimmu.2023.1229562)
Supplement: Supplementary file 1 [file Table_1.pdf]

Supplementary Table 1: Description of the SHIP-TREND-0 study sample.

			HLA-typed	
		**Overall**	**DKMS[1]**	**WGS[2]**
N		976	574	402
Females	N (%)	544 (55.7)	325 (56.6)	219 (54.5)
Males	N (%)	432 (44.3)	249 (43.4)	183 (45.5)
Age	mean (SD)	50.0 (13.7)	45.3 (14.9)	56.7 (7.6)
	range	20-81	20-81	44-70
BMI	mean (SD)	27.3 (4.6)	26.8 (4.6)	28.1 (4.3)
Smoking status				
Current	N (%)	214 (21.9)	158 (27.5)	56 (13.9)
Former	N (%)	350 (35.8)	178 (31.0)	172 (42.8)
Never	N (%)	412 (42.2)	238 (41.5)	174 (43.3)
S. aureus carrier status				
Non-carrier	N (%)	727 (74.5)	423 (73.7)	304 (75.6)
Carrier	N (%)	249 (25.5)	151 (26.3)	98 (24.4)
Carrier CC30+	N (% among carriers)	48 (19.3)	30 (19.9)	18 (18.4)
Carrier *tst*+	N (% among carriers)	49 (19.7)	30 (19.9)	19 (19.4)

[1] DKMS, SHIP-TREND-0 sub-sample that was HLA-typed via PCR-based sequencing by the German Bone Marrow Donor Center (DKMS)

[2] WGS, SHIP-TREND-0 sub-sample that was HLA-typed based on whole genome sequencing data using the HLA-HD algorithm
