# Supplementary material for: Toxin exposure and HLA alleles determine serum antibody binding to toxic shock syndrome toxin 1 (TSST-1) of Staphylococcus aureus
Source: Front Immunol. 2023 Sep 4;14:1229562. doi: 10.3389/fimmu.2023.1229562 (PMC10507260; doi:10.3389/fimmu.2023.1229562)
Supplement: Supplementary file 2 [file Table_2.pdf]

**Supplementary Table 2: Prevalence of *S. aureus* clonal complexes and *tst* in the SHIP-TREND-0 study sample.**

| CC[1] | Prevalence of CCs among all isolates No. (%) | Prevalence of *tst* gene within CCs No (%)[2] |
|---|---|---|
| *tst*-positive CCs | | |
| CC5 | 9 (3.75) | 1 (1.11) |
| CC22 | 15 (6.25) | 1 (6.66) |
| CC30 | 48 (20.0) | 40 (83.33) |
| CC34 | 4 (1.66) | 2 (50.0) |
| CC395 | 5 (2.08) | 5 (100.0) |
| *tst*-negative CCs | 159 (66.25) | 0 (0.0) |
| All strains[3] | 240 | 49 (20.4) |

[1] Clonal complexes (CCs) were determined by *spa* typing (S1).
[2] *tst* was detected by multiplex PCR (S2).
[3] excluding 9 strains that were not available for *spa* typing and virulence gene profiling
