# Supplementary material for: Toxin exposure and HLA alleles determine serum antibody binding to toxic shock syndrome toxin 1 (TSST-1) of Staphylococcus aureus
Source: Front Immunol. 2023 Sep 4;14:1229562. doi: 10.3389/fimmu.2023.1229562 (PMC10507260; doi:10.3389/fimmu.2023.1229562)
Supplement: Supplementary file 3 [file Table_3.pdf]

Supplementary Table 3: Calculation of an allele dosage of HLA-DQB1 and -DRB1 alleles positively or negatively associated with anti-TSST-1 IgGt antibodies.

HLA*DQB1		HLA*DRB1		allele dosage[1]	N
▽	▽	▽	▽	-4	6
▽	▽	▽	Ø[2]	-3	3
▽	Ø	▽	Ø	-2	136
▽	Ø	Ø	Ø	-1	24
Ø	Ø	▽	Ø	-1	4
Ø	Ø	Ø	Ø	0	421
Ø	△	Ø	Ø	1	117
Ø	Ø	Ø	△	1	2
Ø	△	Ø	△	2	148
△	△	Ø	Ø	2	8
Ø	△	△	△	3	1
△	△	Ø	△	3	22
△	△	△	△	4	21
▽	△	▽	Ø	-1	22
▽	△	▽	△	0	30
▽	△	Ø	Ø	0	6
Ø	△	▽	Ø	0	1
▽	△	Ø	△	1	4

[1] The allele dosage of positively (blue triangle, pointing upwards) and negatively (red triangle, pointing downwards) associated HLA alleles was determined by assigning positively associated alleles the arbitrary value of 1 (DQB1*02:01, DRB1*03:01) and negatively associated alleles the value of -1 (DQB1*05:01, DRB1*01:01), and calculating the sum of these values. Allele dosage groups with case numbers ≤ 20 (marked in gray) were excluded from the correlation analysis in Fig. 6.

[2] Ø depicts any non-associated HLA allele
