# Supplementary material for: Toxin exposure and HLA alleles determine serum antibody binding to toxic shock syndrome toxin 1 (TSST-1) of Staphylococcus aureus
Source: Front Immunol. 2023 Sep 4;14:1229562. doi: 10.3389/fimmu.2023.1229562 (PMC10507260; doi:10.3389/fimmu.2023.1229562)
Supplement: Supplementary file 5 [file DataSheet_1.pdf]

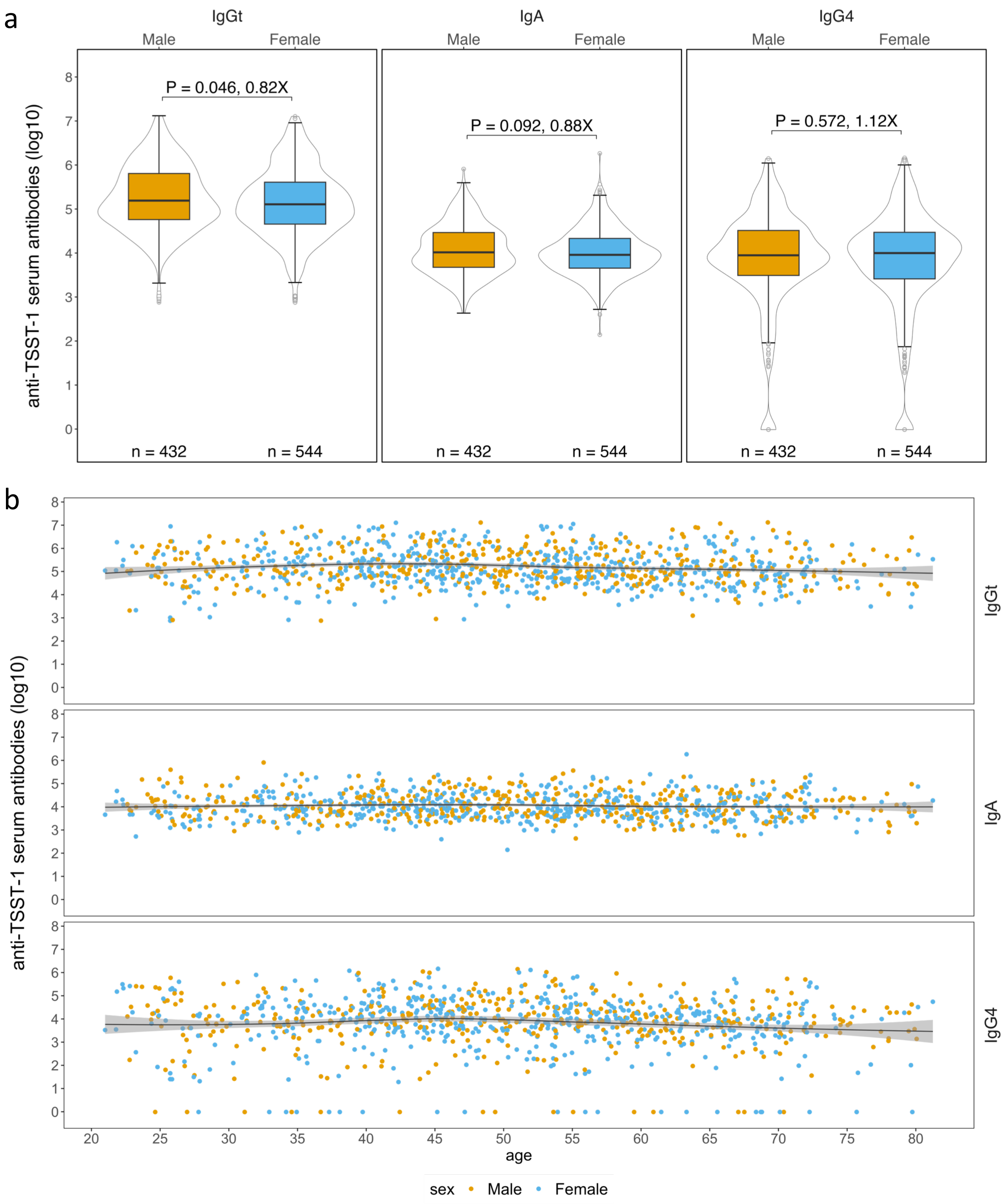

Supplementary Fig. 1: Anti-TSST-1 serum antibody levels stratified by sex and age. Anti-TSST-1 IgGt, IgA and IgG4 antibody levels were quantified using the xMAP® technology and stratified by sex (a) and age (b)(S3). Box plots display median along with the 25 and 75 quartiles, whiskers depict the 25th quantile minus 1.5*IQR and 75th quantile plus 1.5*IQR. Probability density shown as violin plots. Statistics: unpaired Wilcoxon rank sum test (A), non-linear regression using LOESS function (B); X, fold-change between median values.
