# Supplementary material for: Toxin exposure and HLA alleles determine serum antibody binding to toxic shock syndrome toxin 1 (TSST-1) of Staphylococcus aureus
Source: Front Immunol. 2023 Sep 4;14:1229562. doi: 10.3389/fimmu.2023.1229562 (PMC10507260; doi:10.3389/fimmu.2023.1229562)
Supplement: Supplementary file 6 [file DataSheet_2.pdf]

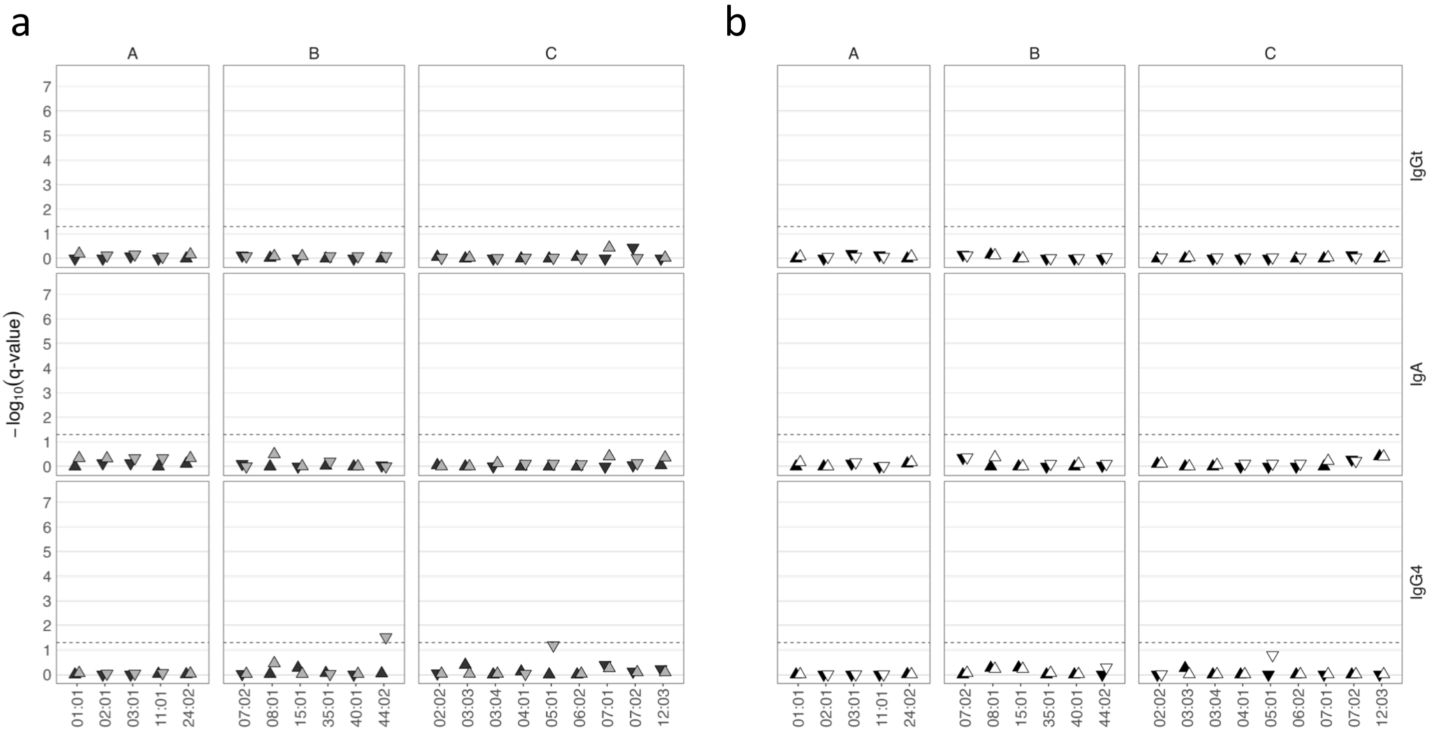

Supplementary Fig. 2: HLA class I alleles are not associated with anti-TSST-1 IgGt, IgA and lgG4 antibody levels. a) A sub-sample of 574 SHIP-TREND-0 participants was HLA-typed by amplification and sequencing of the HLA coding regions (DKMS, light gray). The most frequent HLA class I alleles (frequency ≥ 3%) were correlated with the anti-TSST-1 antibody titers. Findings were validated by deducing HLA haplotypes from whole genome sequencing data using the HLA-HD algorithm (n=402, dark gray). b) Meta-analysis of both approaches (n=976, black). DNA array-based HLA allele prediction using four-digit multi-ethnic HLA v2 imputation panel algorithm (n=965, white). Anti-TSST-1 IgGt (top panel), IgA (middle panel), and IgG4 (bottom panel) levels were not correlated with allelic variants in the HLA class I locus. –log10(q-value), negative decadic logarithm of the Benjamini-Hochberg (BH) adjusted association p-value
