# Supplementary material for: Toxin exposure and HLA alleles determine serum antibody binding to toxic shock syndrome toxin 1 (TSST-1) of Staphylococcus aureus
Source: Front Immunol. 2023 Sep 4;14:1229562. doi: 10.3389/fimmu.2023.1229562 (PMC10507260; doi:10.3389/fimmu.2023.1229562)
Supplement: Supplementary file 7 [file DataSheet_3.pdf]

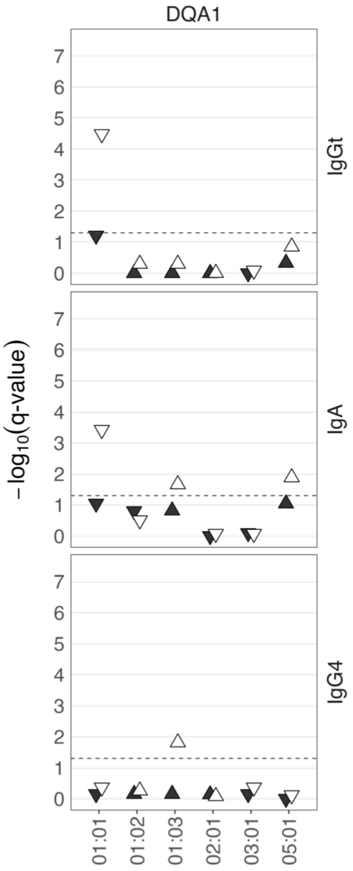

Supplementary Fig. 3: HLA-DQA alleles are associated with low or high anti-TSST-1 IgGt and IgA antibody levels. HLA-DQA alleles were deduced from Illumina WGS data using the HLA-HD algorithm (n=402, dark gray) or predicted from genotyping array data using the Four-digit Multi-ethnic HLA v2 imputation panel (n=965, white). –$\log_{10}$(q-value), negative decadic logarithm of the Benjamini-Hochberg (BH) adjusted p-value.
