# Supplementary material for: Toxin exposure and HLA alleles determine serum antibody binding to toxic shock syndrome toxin 1 (TSST-1) of Staphylococcus aureus
Source: Front Immunol. 2023 Sep 4;14:1229562. doi: 10.3389/fimmu.2023.1229562 (PMC10507260; doi:10.3389/fimmu.2023.1229562)
Supplement: Supplementary file 8 [file DataSheet_4.pdf]

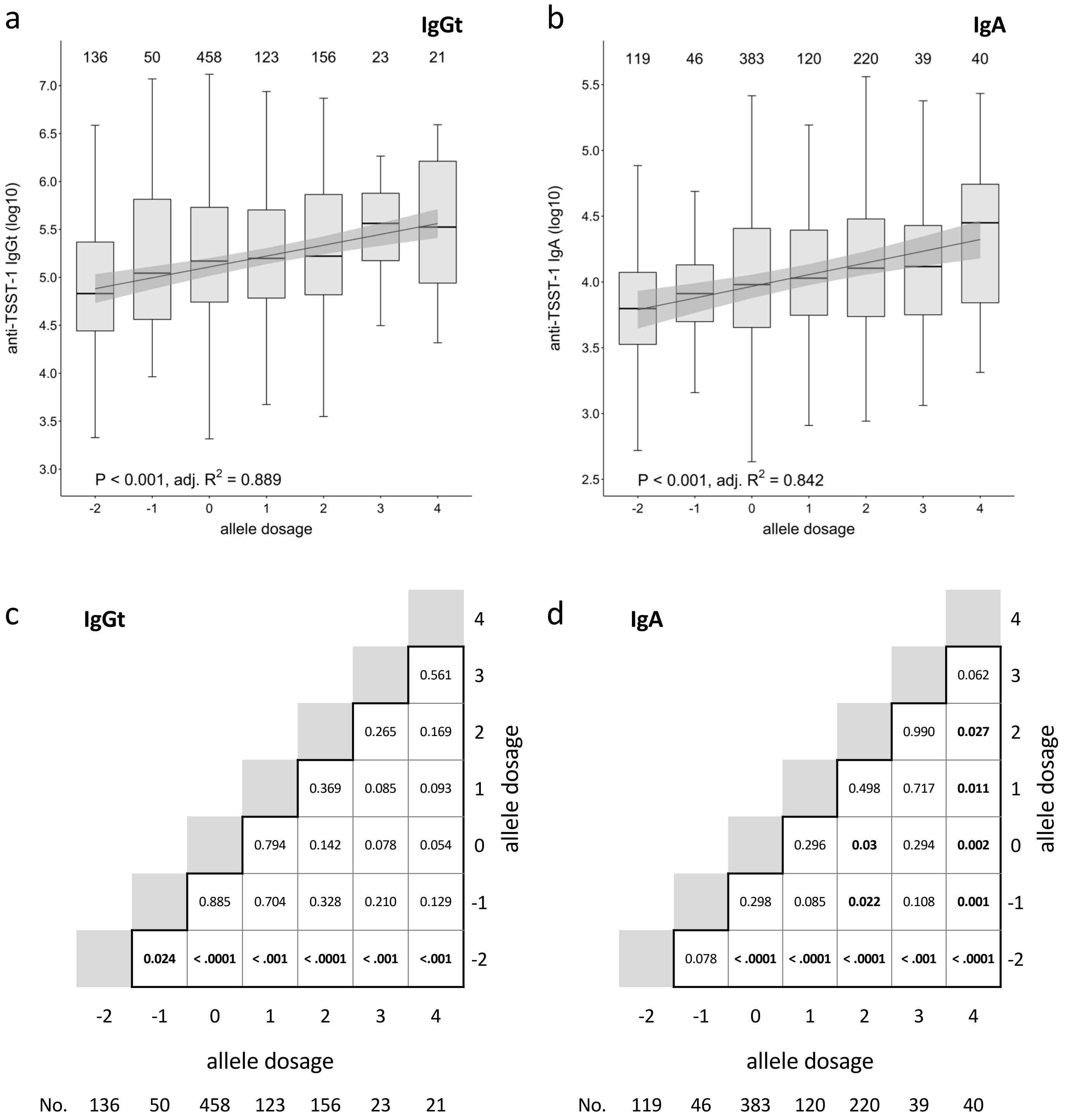

Supplementary Fig. 4: Cumulative effect of positively and negatively associated HLA alleles on anti-TSST-1 IgGt and IgA levels including p values. Allele dosage effects of positively and negatively associated HLA DQB1 and -DRB1 alleles on anti-TSST-1 IgGt (a) and IgA (b) were assessed by assigning positively associated alleles the arbitrary value of 1 (IgGt: DQB1*02:01, DRB1*03:01; IgA: as before, additionally DQB1*06:03 and DRB1*13:01) and negatively associated alleles the value of -1 (IgGt and IgA: DQB1*05:01, DRB1*01:01). The allele dosage was defined as the sum of those values. Pairwise comparisons were performed using the pairwise Wilcoxon rank sum for all combinations of allele dosage groups for anti-TSST-1 IgGt (c) and IgA (d). Significant differences are highlighted in bold letters.
